# Dimensional Structure of MAPS-15: Validation of the Multidimensional Academic Procrastination Scale

**DOI:** 10.3390/ijerph20043201

**Published:** 2023-02-11

**Authors:** Marcela Paz González-Brignardello, Ángeles Sánchez-Elvira Paniagua

**Affiliations:** Department of Personality Psychology, Psychological Assessment and Treatment, Faculty of Psychology, Universidad Nacional de Educación a Distancia (UNED), 28040 Madrid, Spain

**Keywords:** academic procrastination, dimensionality, self-regulation of learning, cross-validation, SEM

## Abstract

Academic procrastination is a complex behavior that hampers the cyclical process of self-regulation in learning, impeding the flow of actions necessary to achieve the goals and sub-goals that students have set out to attain. It has a high frequency of occurrence and has been linked to lessened student performance and a decrease in psychological and physical well-being. The objective of this study is to analyze the psychometric characteristics of a new academic procrastination scale MAPS-15 (Multidimensional Academic Procrastination Scale) applicable in self-regulated learning environments through a cross-validation study (exploratory factor analysis and confirmatory factor analysis). The sample consisted of 1289 students from a distance/online university, with a wide age range and sociocultural variability. The students completed self-reported online questionnaires on two dates: during the university access and adaptation phase and before the first period of compulsory exams. One-, two- and three-factor structures were tested as well as a second-order structure. The results support a three-dimensional structure of MAPS-15: core procrastination, a pure dimension of procrastinating behavior and difficulty in carrying out the action; poor time management, a dimension related to time organization and perceived control over time; and work disconnection, a dimension conceptually related to lack of persistence, and work interruptions.

## 1. Introduction

The concept of lifelong learning arises in response to the growing demands of a highly technological and constantly changing society. People today are undergoing a continuous process of adaptation and renewal of their competencies, which requires frequent participation in training processes. The needs of society and the strong growth and implementation of technology have given way to the development of technology-mediated distance education: online education or e-learning, whose beginnings can be found in the early 1990s, in connection with the expansion of the Internet [1]. Technologies are the distribution channel of learning content and activities in the “online learning environment” [2] a model of teaching and learning, independent of time and space, constantly developing and increasingly accessible and open (see the development of massive open online course—MOOC; the nano open online course—NOOC; or self-paced open online course—SPOOC). However, despite the great strength of online and distance learning—its enormous capacity for distribution and access—it also has the highest dropout rates [3,4]. Being a good learner in e-learning environments, able to initiate and maintain learning processes, seems to require a key competency, that of self-regulated learning; therefore, research on its relationship with dropout or retention in studies is of special interest [5].

Distance-learning environments require learners to be able to self-regulate each of the formative events or episodes that are part of the frequently long path towards the goal; controlling their thoughts, behaviors, emotions, and motivation, in order to achieve the objectives they have set for themselves [6,7]. Furthermore, training in virtual environments “involves temporally delimited processes, strategies, or responses that students must initiate and regulate proactively” [8]. 

Zimmerman and Moylan [9] described a three-phase cyclical model to explain autonomous and self-regulated learning, each phase consisting of different specific strategies: (a) the forethought phase: in which the learner analyzes the task, assesses their ability to perform it successfully, sets goals and plans; (b) the execution and self-monitoring phase, where they self-observe and compare the quality of the ongoing learning process and implement self-monitoring strategies aimed at maintaining concentration and interest; and (c) the self-reflection phase in which the learner judges their work and the results obtained, setting in motion two categories of processes: self-judgment, in which they self-evaluate and make causal attributions, together with self-reaction, i.e., the emotional and cognitive response to his or her own attributions. This cyclical process, whose phases are fed by the products of the previous one, allows the changing of what does not work and the strengthening of useful strategies, and can be interrupted or hindered in any of its stages due to the failure to implement any of the necessary strategies.

Procrastination is one of the disruptive behaviors in the learning cycle. Procrastination is a complex phenomenon involving the interaction of behavioral, cognitive, and emotional components, the theoretical understanding of which has not yet been fully developed [10]. There is consensus that academic procrastination can be understood as a failure in the self-regulation process [10,11,12], although how this relationship occurs is not fully defined. From this perspective of self-regulated learning, a comprehensive approach has been exposed: the temporal motivation theory (TMT) [10,13,14]. This theory supports the view of procrastination like a failure of self-control [15] and integrates three attitude components (affective, behavioral and cognitive) [14].

Its high frequency of occurrence, especially in higher education, has triggered many studies that since the 1990s have been devoted to understanding, measuring, and intervening in this behavior.

One of the most commonly used definitions is that procrastination is the irrational, unnecessary, and involuntary postponement of a planned course of action despite the negative consequences that such a delay will eventually have [10,16,17,18]. It is a behavior that displays the contradiction between the desire to achieve a goal and the lack of persistence in carrying out the necessary steps to achieve it. 

Consequently, a large body of research has developed around the identification of the reasons that lead to procrastination. It has been presented as: a coping strategy for various fears, of failure, success and separation [19]; a mechanism to protect vulnerable self-esteem [20]; avoidance of unpleasant tasks [21] or search for perfection [22]; and a behavior aimed at optimizing time and resources to accomplish a task [23]. Others have considered it a means to increase arousal levels [24]. These two perspectives, that of dysfunctional procrastination [17] and the other, voluntary, intentional, and strategic procrastination [15,25,26], which could be a prioritization behavior fuel an open debate on the functionality or dysfunctionality of procrastination behavior.

Although procrastination manifests itself in different domains: academic [25,26,27], work [28,29,30], financial [31,32], retirement-related [33,34], medical [35,36]; or in habits such as going to bed [37,38], among others; procrastination in academic settings is the most studied. 

Studies of university populations show that the frequency of occurrence of procrastination fluctuates between 23 and 95% [21,39,40,41]; however, more than 70% of university students consider themselves to be procrastinators [42] and 50% procrastinate consistently and problematically [43]. In this area, procrastination is related to student drop-out [44], poor performance [45,46,47,48,49], high stress levels [49,50], lower life satisfaction [51], a deterioration in health and well-being [36], high levels of anxiety [52,53], and depression [54,55].

Many instruments used to measure academic procrastination usually contain questions referring to behaviors observable in face-to-face education or related to tasks or activities typical of this modality of study and which may not be clearly defined in non-face-to-face education. Authors, such as Milgram and Toubiana [56]. have advocated for the need to maintain three main academic tasks that occur in most degrees and at university: homework, exams and writing papers, to consider that an instrument truly captures the characteristics of academic procrastination. For example, several questionnaires contain questions on class attendance: Aitken’s Procrastination Scale [57], Busko’s Student Procrastination Scale [58], McCloskey’s Academic Procrastination Scale [59], de Milgram’s Academic Procrastination Student Form [56,60]; about tasks of attending meetings with professors (Solomon and Rothblum’s Procrastination Assessment Scale-Students [21]; or referring to specific tasks in an area of study, such as making summaries or reference lists as Milgram’s Academic Procrastination Student Form [56,60]. In view of the above, it seems necessary to develop a scale of academic procrastination applicable in different learning environments (face-to-face, virtual or blended learning), and also independent of the area of study and that does not make references to specific academic tasks, being able to be applied to students of science, humanities, technology, etc.

The dimensionality of the construct has been a task of great interest to researchers, and there is currently no consensus on its components [61,62,63]. Ferrari [20] described a model of general procrastination, later widely used, composed of three dimensions: arousal, avoidance and decisional. On the other hand, Steel [64] joined five scales of general procrastination and obtained a unidimensional model and, by forcing it to three factors, failed to replicate Ferrari’s model. Díaz-Morales et al. [62] joined three scales and performed a principal component analysis obtaining four factors: procrastination behavior, lack of punctuality, lack of planning and indecisiveness. 

All of the above shows that there is still not a solid body of knowledge about general procrastination, nor about academic procrastination; knowing that both may not be equivalent. The academic environment presents some characteristics of tasks that are not observed in daily life, tasks susceptible to being postponed and therefore to present procrastination. In the academic entvironment, the permanent evaluation of tasks to which students are subjected to is probably a differentiating element that is not observed in procrastination to go to bed [65], for example. 

It seems necessary to look for more comprehensive assessment instruments that are specifically applicable to the academic environment but are not dependent on the area of study or learning modality.

The main aim of this study is to analyze the structure and psychometric properties of the new Multidimensional Academic Procrastination Scale, MAPS-15. The preliminary version of the scale has previously been used as an experimental instrument [66] without deeply analyzing the dimensional structure. Thus, the following secondary objectives will be set: (a) to extract the factor structure of the scale through exploratory methods (not very restrictive); (b) to confirm the fit of the structural and measurement model of the instrument obtained by means of more restrictive comparison methods (a y b constitute a cross validation method); and (c) to analyze the psychometric properties of reliability and validity of the instrument obtained.

## 2. Materials and Methods

- Participants

The sample for this study was selected on a non-probabilistic purposive basis from freshmen at the UNED (Spanish National Distance Education) with a total of 1406 students. Participants who did not respond to all items (83 students, 5.9% of the sample) were eliminated from the sample. The eliminated data were checked for significant differences (n. s. 95%) in age *(t*_(1305)_ = −0.56; *p* = 0.58); gender *(x*^2^_(1)_ = 1.22; *p* = 0.27); department/school *(x*^2^_(10)_ = 11; *p* = 0.31); prior education *(x*^2^_(6)_ = 5.82; *p* = 0.44) and employment status *(x*^2^_(7)_ = 6.86; *p* = 0.44) against the total sample. In addition, analysis of the pattern of occurrence of missing values showed that they responded to a random structure. We also eliminated those considered multivariate outliers—a total of 34—detected with the Mahalanobis distance (*p* < 0.001), as indicated by Pérez and Medrano [67]. The final total sample was 1289 students, with a mean age of 33.5 and a SD of 9.26; 56.5% were women. In relation to the distribution related to previous studies or form of entry to university (prior education), it was observed that 25.8% came from vocational training (equivalent to higher technical schools), 20.2% through university entrance exams, 19% from university access courses for students over 25/45 years of age, 15% had a university degree, 12.8%; had a bachelor’s degree (three years) and 0.4% had a Ph.D. (the rest, 6.3%, did not answer the question). All faculties and schools of the university were represented in the sample, with most students from Economics (19.2%), Psychology (19.0%), and Law (18.5%). The remainder was distributed among the other faculties and schools. Most of the participants were ‘employed workers’, 53.1%, followed by students who were ‘unemployed with previous work experience’, 20.2%, and ‘only students’ at 9.9%. The remainder was distributed among other classifications: ‘self-employed’, 4.9%, ‘unemployed without previous work experience’, 2.2%, ‘unpaid domestic work’, 1.7%, and ‘retired’, 0.8% (6.6% did not answer this question).

- Instruments
  - The Academic Procrastination Scale, a preliminary version consisting of 18 items. Seven items from the Low Work Discipline subscale of the Schouwenburg Study Problems Questionnaire (SPQ) [68] were selected. This questionnaire was created by Hermans in 1977 (unpublished manuscript cited in Hughes [69]). This subscale (initially called Work Discipline) represents the perceived ability to resist procrastination while studying. One item from the Decisional Procrastination Questionnaire (DP; Man, 1982, cited by Díaz-Morales et al. [62]) related to conflicting decisions was selected and adapted. Two other items from the Procrastination subscale of the Academic Procrastination State Inventory (APSI; Schouwenburg, 1992, 1995) [29] were selected and adapted too. Two items from the General Procrastination Scale (GPS; Lay, 1986, cited in Díaz-Morales et al. [62]) were adapted. Six other items were created based on the authors’ teaching and tutorial experience in distance-learning environments and self-regulated learning. The selection process was carried out collaboratively by the authors based on an analysis of the content of each item: the items most representative of academic procrastination behavior understood as difficulty in initiating, maintaining, or completing the performance of learning and study tasks. We have also evaluated the applicability of the items to the e-learning environment, their applicability to any area of study (engineering, sciences, humanities, etc.) and to any learning style or strategy. The items were translated from English into Spanish and two psychologists, with extensive experience in the prevention of student drop-out in distance higher education, checked the content and face validity of the 18 items that made up the initial scale. In the case of disagreement, the wording was adapted until full agreement was reached. Finally, the items were presented in a 5-point Likert response format measuring the degree of agreement with the proposed statements, with extreme values of 1 = not at all and 5 = completely (Appendix A). Items 9, 10, 14, and 15 are reverse items included in the scale following the guidelines of Navas [70]. Higher scores reflect greater academic procrastination.
  - The Time Management Behavior Questionnaire, TMBQ, of Macan [71,72], Spanish version of García-Ros and Pérez-González [73]; is a self-administered instrument with 34 items related to learning time management. Student responses indicate the degree to which each item describes the usual way of managing time, using a 5-point Likert-type response scale, where 1 corresponds to “never” and 5 to “always”. The instruments is composed of four dimensions: (a) establishing objectives and priorities, evaluating the capacity to select and prioritize academic tasks (e.g., “Divide complex and difficult projects into smaller more manageable tasks”); (b) time management tools, assessing the use of techniques related to effective time management, as to schedule or use of the agenda (e.g., “I make a list of the things I have to do every day and put a mark next to each task when I have finished it”); (c) preferences for disorganization, evaluating the degree of prior planning and structuring, as well as the maintenance of a disorganized study setting, (e.g., “My work days are too unpredictable to plan and manage my time”); and d) perception of control over time, evaluating the degree of perceived control over time management, as well as the lack of control (e.g., I have to spend a lot of time on unimportant tasks”). García-Ros and Pérez-González [73] reported the following reliability indices (Cronbach’s alpha) for each dimension: (a) 0.84; (b) 0.79; (c) 0.71 and (d) 0.72 obtained in a Spanish sample.
  - Short ad hoc questionnaire on socio-demographic information made up of closed questions to collect data on age, sex, studies prior to entering university, and current occupation. The data concerning the faculty/school were collected automatically and internally on the e-learning platform where the instruments were presented.
- Procedure

All incoming freshmen and first-year students that enrolled at the university were invited to participate in an institutional program aimed at learning about the characteristics and needs of incoming students. This invitation was made within the virtual induction communities (part of the institutional welcome program) through the university’s e-learning platform, making use of its communication tools (e-mail and forums). 

The program consisted of eight online information records, through the university’s educational platform and within the host communities, during the first semester of studies. The data presented in this paper refer to records number 2 (entry and adaptation phase) and number 6 (prior to the first period of mandatory exams). Students who completed all phases of the study received one free configuration credit. Explicit and voluntary informed consent was requested.

- Data analysis

For purposes of cross-validation, the total sample of 1289 participants was randomly divided into two, resulting in a sub-sample A of 632 individuals for the exploratory factor analysis (EFA) and a sub-sample B of 657 for the CFA. The equivalence of both sub-samples was studied (*p* < 0.05) in the following socio-demographic variables: age, through a *Student’s t-test* for mean differences (M_EFA_ = 33.34 and SD_FA_ = 9.24; M_CFA_ = 33.75 and SD_CFA_ = 9.30; *t*_(1206)_ = −0.76 and *p* = 0.45). The grouping variables were compared through a test of independence, *X*^2^ test, giving the following results: gender (Women_EFA_ = 57.3%; Women_CFA_ = 55.9%; *x*^2^_(1)_ = 0.30 and *p* = 0.58); distribution of students according to previous level of studies before university entrance (*x*^2^_(6)_ = 0.97 and *p* = 0.99), according to employment status (*x*^2^_(7)_ = 13.37 and *p* = 0.06), and faculty/school (*x*^2^_(10)_ = 4.32 and *p* = 0.93). Both sub-samples could be considered equivalent *(p* < 0.05). The estimate method used in analyses was *robust unweighted least squares* (*RULS*) [74] due to the presence of high multivariate kurtosis informed by the Mardia analysis [75]. 

### 2.1. Exploratory Factor Analysis (EFA)

To study the factorial structure of the scale, an EFA was carried out with the FACTOR 10 software [76]. The matrix of polychoric correlations [77] between the 18 items of the scale was obtained and the suitability of the data to factorization was determined through the Kaiser–Meyer–Olkin index (KMO) and Bartlett’s sphericity test. 

Three EFAs were carried out to explore the one-, two- and three-factor structure and the *Promin* oblique rotation method was used to interpret the solutions obtained [78,79]. Items were retained if their saturation was ≥ 0.40 [80] and they did not present factor loadings equal to or higher than this value in two or more factors of the same solution simultaneously [81]. To determine the number of factors, we followed the recommendations given by Ferrando and Anguiano-Carrasco [78] based on the use of multiple indicators: the parallel analysis (PA) based on *minimum rank factor analysis* (MRFA), and the value of the cumulative variance extracted. In addition, the software used made it possible to obtain statistical indices of goodness-of-fit [82], which allowed the following indicators of each dimensional structure to be known and compared: the *root-mean-square of residuals* (*RMSR*), the *root-mean-square error of approximation* (RMSEA), the *goodness-of-fit Index* (GFI), and the *comparative fix index* (CFI) [83]. RMSEA values ≤ 0.05 indicate good fit and their 90% confidence interval (CI) lies within the 0 and 0.05 range, values between 0.08 and 0.10 provide a mediocre fit, while values above 0.10 indicate a clear misfit of the data. CFI and GFI values ≥ 0.95 indicate a very good fit of the model to the data. For RMSR a good fit is considered to be indicated by values ≤ 0.05 [84]. 

Following Thurstone’s proposal that “the essence of FA is to determine the minimum number of common factors that is compatible with acceptably low residuals” (cited in Ferrando et al., p. 13 [74]) we proceeded to analyze the standardized residuals matrix. In the literature, a value of |2.58| corresponding to the level of α = 0.01 has been defined as a criterion.

The reliability coefficient of each structural solution was obtained through the αordinal for each factor [85] and the coefficient of determination (R^2^), which is interpreted as the proportion of the variation that can be explained by each dimension. The calculation of the correlation between factors for each solution allowed information on the discriminant validity of each factor to be collected.

Once the structure was defined with greater statistical support, better goodness-of-fit, less unexplained residuals, and with good reliability and validity indices, we proceeded to name the factors based on the content of the items with the highest saturation in the factor.

### 2.2. Confirmatory Factor Analysis

To test and replicate the solutions obtained and identified in the exploratory analysis, CFAs were performed under more restrictive statistical assumptions [86]. Given that the theoretical models for procrastination do not fully define the one- or multidimensionality of the construct, and with the aim of investigating the behavior of the scale, we proceeded to compare the one-factor structure (supported by the PA in the EFA) with the three-factor structure, which also performed well statistically (especially in terms of the fit indices and residuals analysis). As a third option, a second-order-integrated structure was studied to test whether a model based on a general factor could explain the dimensional organization of the scale. The CFAs were performed on the data from sub-sample B. The robust unweighted least squares (*RULS*) estimation method was used, a method that uses the polychoric correlation matrix as a starting point to subsequently obtain the asymptotic covariance matrix [87]. 

Multivariate normality compliance analyses were performed using Mardia’s index and show that the multivariate kurtosis of the data for sample B was, at a significance level of 0.05%, different from a multivariate normal distribution (kurtosis coefficient = 434, test statistic = 36.1 and *p* < 0.001); while the skewness coefficient was 27.1, statistic = 3074 with 1140 degrees of freedom and *p* = 1.000. A kurtosis significantly different from normal affects the validity of the results more than a non-normal multivariate skewness value as discussed in González et al. [88], citing Bollen. Given this situation, it was decided to use the RULS estimation method [89] suitable for ordinal values and robust in the case of non-compliance with multivariate normality.

The goodness-of-fit assessment was carried out using a combination of absolute and relative fit indices [90,91]. Among the absolute indices, the Satorra–Bentler *X^2^* (*X*^2^_SB_), a corrected index for non-normal samples [92], was used to test the hypothesis that the variance–covariance matrix reproduced by the model was equal to the observed matrix. The ratio of *X*^2^_SB_ to the degrees of freedom (*X*^2^_SB_*/df*) was reported. Other goodness-of-fit indices were also reported in a complementary manner: RMSEA, GFI, CFI, and RMSR. 

### 2.3. Fiability and Validity 

Once the best fit of the three-factor model was confirmed, the reliability and validity of the test was calculated. The analysis of the individual parameters was carried out through the proportion of variance of each item explained by the factor to which it belongs, for which the individual reliability (λ^2^) was calculated with a value ≥ 0.25 [93] being suitable. Then, to study the behavior of each factor, the internal consistency was calculated through the composite reliability coefficient (CFC) and the average variance extracted (AVE). Both indices are calculated from the factor loadings and the error variance of the items that make up the factor [94]. Classical criteria indicate that adequate construct reliability corresponds to CFC ≥ 0.70 and an AVE ≥ 0.50 as an indicator of satisfactory convergent validity [93]. However, Moral de la Rubia (2019) [95] questioned the criterion used for AVE given the influence that the number of items has on it in structural equation models. The author proposed the use of less restrictive criteria to consider an acceptable level of convergent validity, if factor loadings λ ≥ 0.50, individual reliability values λ^2^ > 0.25 and Omega reliability values (ω) or H ≥ 0.70 are maintained. 

The temporal stability of the factorial solution was studied, by means of a test–retest at two months, based on the intraclass correlation coefficient (ICC) that allows measuring the concordance of the score at two different temporal moments [96,97] in our case, a test–retest procedure at 3 months. A mixed model with absolute concordance was chosen, as it tests the equivalence of the measures observed at both time points. It is considered a good indicator of stability if the ICC value > 0.70 and excellent if >0.90. 

Evidence of concurrent validity was sought through the correlations of the MAPS-15 with the Time Management Behavior Questionnaire (TMBQ) [72] and, based on the results obtained by Garzón Umerenkova and Gil Flores [98], it was expected to obtain high correlations, in a negative sense, with the goal and priority setting subscale and with perception of control over time; both scales related to elements of self-regulation that may underlie procrastination; therefore, we expect high or moderate correlations with any of the dimensions of MAPS-15. By the other hand, in relation to disconnection, we would expect to find a significant and positive relationship with preference for disorganization. At the same time, we would expect to find, in general, low, or null correlations with time management tools. 

The descriptive analyses of this study were performed with SPSS© v.25; FACTOR 10 [76] for EFAs and LISREL 8.8 for Windows© was used to perform the CFAs; Excel© (to calculate reliability and validity) and the Domínguez-Lara’s [99] specific module to calculate the ordinal α were also used.

## 3. Results

### 3.1. Extraction of the Factor Structure (EFA)

The data adequacy for using the factor analysis technique was obtained through the Kaiser–Meyer–Olkin measure (KMO = 0.93) and Bartlett’s sphericity statistics (3663 with 153 *df*, *p* < 0.001); values that supported the factorization of the data. 

We started by extracting a one-dimensional factor structure, which yielded 40% of the explained variance. Parallel analysis (PA) indicated the existence of a single dimension but given the small amount of explained variance, two and three factors were also extracted, which cumulatively explained 47 and 53% of the variance. The factor loadings obtained for each of the extracted rotated models (*Promin*) are shown in Table 1. All items had loadings that exceeded the criterion value of ≥0.40 except item 18 in the unidimensional solution; items 1, 16 and 18 in the two-factor solution; and items 1, 13, and 18 in the three-factor solution, which were eliminated from the respective models. Regarding item ambiguity, item 4 presented similar loadings in two factors of the three-factor solution (F1 and F3); however, we decided to keep it in factor three because its content coincides with the general content of the factor and gives meaning to the dimension. A new AFE was performed for each structure, this time without the deleted items, obtaining an explained variance of 42% for the single-factor structure, 52% for the two-factor structure, while the three-dimensional model explained 59% of the variance (final solution in Table 1).

Next, Table 2 shows the goodness-of-fit indices, which allow comparing the behavior of the three solutions. The RMSEA value of the three structures was within the range of values indicative of good fit (<0.06) with the value of the three-factor solution being the best, with a wide CI range <0.05. The GFI and CFI of the three solutions gave values indicating a very good fit (>0.95). As for the RMSR value, the three-factor model met Kelley’s criterion (equal to or less than 0.040 for the N of this sample) which is the recommended mean for an acceptable model: “If the RMSR is around this value, or is lower, it can be interpreted that the observed residual values are not significantly different from zero and, therefore, that there are no systematic relationships left to explain” [78].

The ordinal α for each factor, in each extracted solution, showed values ≥ 0.70 indicating that the level of reliability (internal consistency) in all models was good or sufficient [100], except for F3 of the three-dimensional structure, which showed a value slightly below the criterion. The inter-factor correlation of the two-factor solution was r_F1/F2_ = 0.81 and R^2^_F1/F2_ = 0.66 which suggests that both factors are measuring closely related aspects of the overall construct, while the correlations and coefficients of determination of the three-factor model indicated that each factor adds specific information to a greater extent to the understanding of the multidimensional construct (r_12_ = 0.66, R^2^_12_ = 0.44; r_13_ = 0.74, R^2^_13_ = 0.55; r_23_ = 0.66, R^2^_23_ = 0.44), providing evidence of greater discriminant validity. On the other hand, the degree to which the factors were found related to each other could indicate that they belong to a higher-level common factor in a second-order structure.

The identification of extreme standardized residuals (>|2.58|) gave the following result: (a) for the one-dimensional structure, 5 negative and 13 positive residuals were identified, with median residuals = −0.13; (b) for the two-dimensional structure, two negative and four positive residuals, with median = −0.12; and (c) for the three-factor structure, only one larger positive residual was identified, with median = −0.05. 

Once the three-factor solution was preferentially supported, the name was assigned according to the content of the items with the highest loadings in each factor: Factor 1: core procrastination (CP), consisting of items 5, 6, 7, 8, 11, and 15, with item 11 ‘*I usually postpone studying because I think I still have a lot of time left to do it*’ and item 8 ‘*I find it difficult to make the decision to start studying*’; the most saturated items in it; Factor 2: poor time management (PTM), consisting of items 3, 9, 10, and 14; with item 10r ‘*I keep on top of my studies*’ (reverse item) having the highest loadings; and Factor 3: work disconnection (WD), consisting of items 2, 4, 12, 16, and 17, with item 17 being the item saturated with the most weight in the factor: ‘*When I’m studying I waste a lot of time on irrelevant information before I get down to the main ideas of a subject*’.

### 3.2. Confirmatory Factor Analysis (CFA) 

The polychoric correlation matrix was created, which is the basis of the estimation method to use *(RULS*) and on which the asymptotic covariance matrix was generated for the sample B. The matrix of polychoric correlations did not present values outside the range ±1, nor negative variances, and was positively defined, valid for the CFA. 

In this phase of the cross-validation process, five models were compared: the three-factor model identified as the model with the best indicators in the previous EFA; the one-factor model that is supported by part of the literature; the two-factor model (partially supported by the EFA) and two second-order models that were proposed to address the high correlations between factors or lack of discriminant validity in the two- and three-factor structures. The results of the CFAs can be found in Table 3. The *X*^2^ values indicate that all five models, with a *p* < 0.001, were unlikely and should be rejected. The X^2^_SB_ value was also reported as a better fit to the type of data and the non-normality of the sample and the ratio of *X*^2^_SB_/*df* was obtained. All models obtained a ratio <5.0, indicating an adequate fit [93]; however, the three-factor model was close to the most stringent criterion, indicating a good fit. Various goodness-of-fit indices were calculated and thus allowed comparing the models and identifying the model with the best possible fit [93]. 

As can be seen in Table 3, the three-factor model presented the best indicators compared to the other four models; although all of them presented adequate or very good values for RMSEA, CFI and GFI. Only the two- and three-factor models presented an adequate value below 0.5 in SRMR.

The three-factor, 15-item scale, finally called the Multidimensional Academic Procrastination Scale-15 (MAPS-15), is the one that presented, among the five models analyzed, the best goodness-of-fit indicators. 

### 3.3. Evaluation of the Reliability and Validity of MAPS-15

In relation to the individual reliability of each item (λ^2^) we can see that all of them met the criterion (≥0.25) except item 12 (Table 4). As for the composite reliability coefficient (CRC), the three factors presented values compatible with adequate reliability (≥0.70) like in the rest of the reliability indicators reported (ω and H). Therefore, it can be stated that the three factors are strongly and stably defined. 

Discriminant validity was studied following the indications of Marôco [93], so that the AVE of each factor or construct must be equal to or greater than the squared correlations (R^2^) between that construct and each of the other factors. The values obtained for bivariate correlations and their R^2^ were: r_(CP-PTM)_ = 0.79 and R^2^_(CP-PTM)_ = 0.62; r_(CP-WD)_ = 0.86 and R^2^_(CP-WD)_ = 0.75 and r_(PTM-WD)_ = 0.79 and R^2^_(PTM-WD)_ = 0.62. We see that the factors of the three-dimensional scale did not discriminate sufficiently. The factors had high intercorrelations with each other, r_(CP-PTM)_ = 0.79; r_(CP-WD)_ = 0.86 and r_(PTM-WD)_ = 0.79; replicating what was already obtained in the EFA and suggesting the existence of a second-order structure. However, this study did not present clear evidence of a better fit than the three-factor structure. 

The factor loadings of each item allowed us to obtain evidence about the convergent validity of the items that make up each factor. In Table 4 we see that the item loadings (λ) mostly had values > 60. Only items 7, 12, and 15 were below the criterion. Moreover, all loadings had *t*-values > 1.96 *(p* < 0.05). The data also allow us to affirm the existence of convergent validity for the poor time management factor, based on the AVE value, as it exceeded the classical criterion level of 0.50, while the core procrastination factor did not reach the criterion but presented a minimal difference from it. The work disconnection factor, however, showed an AVE = 0.41, with the value furthest from the criterion. If more flexible AVE criteria relating to the number of items in each factor are used, convergent validity would be confirmed for all three factors [95], criteria applicable when the requirements for individual item reliability ≥0.25 (except item 12) and for the omega (ω) and H factor reliability indices ≥0.70 are met (see Table 4).

The next step was to calculate the temporal stability. For this purpose, after the first data collection (T1), a second one was performed after 3 months (T2). The intraclass correlation index (ICC_T1/T2_) was measured and the following results were obtained: CP_T1/T2_ = 0.853; PTM_T1/T2_ = 0.855 y WD_T1/T2_ = 0.835; all of them indicative of good temporal stability as they were above 0.70.

The correlations obtained between the MAPS-15 dimensions and TMBQ subscales constitute evidence of the concurrent validity of the instrument under study (c.f. Garzón Umerenkova and Gil Flores [98]). In Table 5 we can observe how the three dimensions of academic procrastination measured by the MAPS-15 correlate with perceived time control (PCT), as well as with establishing objectives and priorities (EOP) both showing large effect sizes. More novel, but expected, is the moderate correlation between preference for disorganization and the work disconnection (WD) dimension.

## 4. Discussion

The main aim of this study was to analyze the dimensional structure of the MAPS-15, an instrument to assess academic procrastination. The results obtained allow us to affirm that the instrument is made up of three dimensions that present a high level of reliability and internal consistency and with adequate convergent validity. At the same time, its structure and composition are invariant in terms of factor structure, item loading and saturation and in relation to measurement errors. The invariance of the variance–covariance matrix of the factors could not be confirmed. The three factors found were: core procrastination (delay in the initiation and development of tasks and difficulty in deciding to start studying); poor time management (difficulty keeping up with studies, falling behind) and work disconnection (disorganization, lack of persistence, interruptions). 

These three factors could be related to the factors obtained by Díaz-Morales et al. [62] when carrying out a principal component analysis on the data obtained from three general procrastination scales in a Spanish sample. The authors found a multidimensional structure consisting of procrastination behavior, which measured the predisposition to manifest intentional behavioral lags; lack of punctuality, as the inability to work diligently to meet task deadlines; lack of planning, which measured the lack of self-discipline to stay on task; and indecisiveness, referring to putting off making decisions by a defined deadline. MAPS-15 collects, in a single instrument, three factors that appear distributed on different scales and does so in a short-scale format. This is probably one of the great contributions of this instrument. The items that make up the MAPS-15 are distributed among the different factors of the scale, independently of their original scale or subscale, i.e., they do not remain linked to each other. The seven items of the procrastination subscale of the SPQ have been distributed among the three factors. The same is true for the other items. This seems to indicate that the MAPS-15 presents a novel and representative conceptual organization of the different theoretical components of the construct–previously found–organized in a new multidimensional perspective, within a single instrument.

On the other hand, academic procrastination has been considered a failure of self-regulation [10,11], a manifestation of a lack of learning regulation skills and strategies. Academic procrastination, measured through the MAPS-15 could reflect difficulties in the set of strategies fundamental to the first two phases of Zimmerman’s self-regulation model of learning: preparation and planning, and execution, in the form of actions opposed to self-regulation strategies: assessment of time and task needs, time planning, lack of persistence, distraction and task avoidance, among others. 

Based on these findings, it is necessary to study the behavior of the different dimensions in relation to other procrastination scales, and within a nomological network that includes self-regulated learning variables and motivational and emotional/affective aspects. This will help to resolve the most controversial aspect of this study: the discriminant validity of the factors. In terms of ecological validity, it is desirable that the self-report measure be related to observational measures of behavior. Although temporal stability has been evaluated and verified, the time interval was small (3 months), and it would be advisable to re-evaluate this aspect with measurements at least at 6 months.

On the other hand, in relation to the predictive validity, an interesting avenue of study is opened on the predictive power of the three-dimensional structure in relation to academic performance and the persistence or attrition of students. 

## 5. Conclusions

In this study we have validated the factor structure of a new instrument: the Multidimensional Academic Procrastination Scale, MAPS-15, using the cross-validation method and structural equation modeling (SEM). In this way, we have been able to verify that the three factors obtained that satisfactorily explain the variability of the data, through a scale that provides reliability, validity and temporal stability. The three factors include: a pure dimension of procrastinatory behavior and difficulty in performing the action (core procrastination); a dimension related to time management, perceived control over time and planning (poor time management); and a dimension conceptually related to the lack of persistence, concentration and organization (work disconnection). 

The knowledge and operationalization of a construct requires the rigorous analysis of the measurement instruments, as a previous step to the association and prediction studies. Structural equation models (SEM) have gained relevance in recent decades as powerful statistical tools that allow the validation of theoretical models in measurement instruments. Based on the covariations of the observed variables, the SEM makes it possible to estimate latent, non-observable constructs, and thus support the reliability and validity studies of the instruments. 

This study allows us to offer a new measurement instrument, usable in different learning modalities, that understands academic procrastination as a complex and multidimensional behavior. The statistical indices and theoretical conceptualization of academic procrastination, in this study, support the three-dimensional structure; however, the statistical indicators do not drastically invalidate the competing models, leaving future research open.

This validation study was conducted with students from a distance-learning university; however, its formulation was not focused on this modality of study. Its items seem to be applicable in face-to-face or adult higher-education environments; but the behavior of the scale for other types of students should be confirmed in the future.

## Figures and Tables

**Table 1:** Factor or configuration matrix—oblique rotation—for 1-, 2- and 3-factor solution.

	One Factor	Two Factors			Three Factors	
Item	F1	F1	F2	F1	F2	F3
item1	0.57	0.25	0. 35	0.17	0.09	0.38
item2	0.68	0.24	0.47	0.16	0.19	0.42
item3	0.68	−0. 18	0.91	−0. 13	0.58	0.36
item4	0.58	0.59	0. 03	*0.43*	−0. 20	*0.41*
item5	0.58	0.56	0. 05	0.49	0. 02	0.13
item6	0.77	0.52	0. 28	0.48	0. 21	0.16
item7	0.50	0.80	−0. 27	0.66	−0. 29	0.16
item8	0.74	0.88	−0. 09	0.81	0. 00	0.00
item9r	0.67	0. 17	0.54	0.27	0.60	−0. 10
item10r	0.66	−0. 16	0.86	−0. 07	0.96	−0. 09
item11	0.70	0.84	−0. 10	0.82	0. 04	−0. 10
item12	0.41	−0.15	0.58	−0. 23	0.18	0.54
item13	0.69	0. 30	0.43	0. 27	0.24	0.26
item14r	0.55	−0. 16	0.75	−0. 12	0.49	0.27
item15r	0.48	0.58	−0. 08	0.65	0.15	−0.28
item16	0.65	0. 39	0. 29	0. 22	−0. 08	0.59
item17	0.57	0. 15	0.45	−0. 06	−0. 04	0.76
item18	0. 09	0. 02	0. 07	−0. 02	−0. 04	0.16
α ordinal	0.90	0.84	0.84	0.82	0.74	0.67
% Expl var i.s./f.s.	0.40/0.42	0.47/0.52			0.53/0.59	

Values that do not meet the load criterion ≥ 0.40 are shown attenuated and ambiguous loads are shown in shaded italics. i.s. = initial solution; f.s. = final solution.

**Table 2:** Goodness-of-fit indices.

Solution	RMSEA	CI RMSEA		GFI	CFI	RMSR *	CI RMSR	
		Low	High				Low	High
1 factor	0.06	0.05	0.06	0.98	0.98	0.06	0.06	0.06
2 factors	0.05	0.04	0.05	0.99	0.99	0.04	0.04	0.04
3 factors	0.03	not calculated		0.99	0.99	0.03	0.03	0.03

CI: 95% confidence interval obtained through resampling (bootstrap). * RMSR < 0.04 (Kelley’s criterion).

**Table 3:** Fit indices for the five factor models.

Model	*X* ^2^	*df*	*X* ^2^_SB_	*X*^2^_SB_/*df*	RMSEA	RMSEA CI	CFI	GFI	RMSR
One-factor	920.10	119	573.50	4.82	0.076	(0.070; 0.082)	0.970	0.986	0.054
Two-factor	579.28	88	356.43	4.05	0.068	(0.060; 0.076)	0.978	0.989	0.043
Three-factor	460.95	87	277.20	3.19	0.057	(0.050; 0.065)	0.984	0.990	0.043
2nd order (2F)	707.91	88	438.29	4.98	0.078	(0.071; 0.085)	0.971	0.987	0.051
2nd order (3F)	666.72	87	411.93	4.73	0.075	(0.068; 0.082)	0.973	0.988	0.051
*x*^2^; *p* < 0.001									

**Table 4:** Factor structure, reliability, and average variance extracted.

	Core Procrastination	Poor Time Management	Work Disconnection			
Item	λ	λ	λ	λ^2^	M	SD
item5	0.61			0.37	1.77	0.95
item6	0.81			0.65	2.16	1.09
item7	0.58			0.34	2.16	1.16
item8	0.77			0.59	2.12	1.05
item11	0.80			0.63	2.26	1.13
item15	0.54			0.29	2.61	0.99
item3		0.72		0.52	2.68	1.02
item9		0.83		0.68	2.71	1.07
item10		0.81		0.65	3.03	1.00
item14		0.61		0.38	2.79	0.96
item2			0.74	0.55	2.23	1.10
item4			0.67	0.45	1.96	0.96
item12			0.44	0.19	3.35	1.17
item16			0.68	0.46	2.33	1.02
item17			0.62	0.39	2.40	1.03
CRC	0.84	0.83	0.77			
AVE	0.48	0.56	0.41			
Index ω	0.84	0.83	0.71			
H	0.89	0.88	0.85			
For all factor loadings *t* > 1.96; *p* < 0.05.						

**Table 5:** Descriptive and correlations (Pearson’s bilateral) between variables.

Subscales	Mean	SD	1	2	3	4	5	6
1. MAPS-15_CP	13.34	4.98						
2. MAPS-15_PTM	11.88	3.67	0.66 **					
3. MAPS-15_WD	12.61	3.86	0.68 **	0.66 **				
4. TMBQ_EOP	42.79	6.31	−0.44 **	−0.49 **	−0.41 **			
5. TMBQ_PCT	20.54	4.59	0.53 **	0.60 **	0.61 **	−0.52 **		
6. TMBQ_TMT	27.12	6.85	−0.27 **	−0.27 **	−0.21 **	0.56 **	−0.24 **	
7. TMBQ_PD	10.66	3.34	0.32 **	0.28 **	0.35 **	−0.27 **	0.38 **	−0.20 **

MAPS-15: Multidimensional Academic Procrastination Scale; CP: core procrastination; PTM: poor time management; WD: work disconnection; TMBQ: time management behavior questionnaire; EOP: establishing objectives and priorities; PCT: perception of control over time; TMT: time management tools; PD: preference for disorganization; ** *p* < 0.01.

## Data Availability

Data can be accessed via contacting the authors upon approval.

## Appendix A

**Table A1:** Multidimensional Academic Procrastination Scale Items (English and Spanish).

Item	Content	MAPS-15 Factor
*Item1*	*I can’t get down to hard work* *(No puedo ponerme a trabajar arduamente)*	*----*
Item2	I work in a non-systematic (disorganized) way. (Trabajo en forma no sistemática (desordenada))	Work Disconnection
Item3	I am always behind with my work (Voy siempre atrasado/a con mi trabajo)	Poor Time Management
Item4	I am always interrupting my work to smoke, have a coffee, walk around, chat with someone… (Continuamente interrumpo mi trabajo para fumar, tomar un café, dar vueltas alrededor, conversar con alguien...)	Work Disconnection
Item5	I do not care enough about my studies (No me preocupo lo suficiente de mis estudios)	Core Procrastination
Item6	I work on and off, when I feel like it (Trabajo irregularmente, por impulsos)	Core Procrastination
Item7	I would like to have a real incentive to work (Echo de menos un incentivo real para trabajar)	Core Procrastination
Item8	I find it difficult to make the decision to start studying (Me cuesta tomar la decisión de ponerme a estudiar)	Core Procrastination
Item9r	I have a study routine that I stick to (Tengo una rutina para estudiar que suelo cumplir)	Poor Time Management
Item10r	I keep on top of my studies (Suelo llevar al día el estudio)	Poor Time Management
Item11	I usually postpone studying because I think I still have a lot of time left to do it (Habitualmente pospongo estudiar porque pienso que aún me queda mucho tiempo para hacerlo)	Core Procrastination
Item12	I usually think that I will be able to do more things in a day than I can actually get done (Suelo tener la sensación de que podré hacer más cosas en el día de las que finalmente puedo hacer)	Work Disconnection
*Item13*	*I tend to study instead of doing things that I might feel like more* *(Suelo ponerme a estudiar más tarde de lo que había planificado o había previsto)*	*----*
Item14r	I usually manage to study everything I set out to study for a session (Habitualmente consigo estudiar todo lo que me había propuesto para una sesión)	Poor Time Management
Item15r	I tend to study instead of doing things that I might feel like more (Suelo ponerme a estudiar, en vez de ponerme a hacer cosas que pueden ser más apetecibles)	Core Procrastination
Item16	When I’ve been studying and prepping for exams, I wasted time doing other things (Cuando he estado estudiando y preparando los exámenes he perdido el tiempo haciendo otras cosas)	Work Disconnection
Item17	When I am studying, I waste a lot of time on irrelevant information before I get down to the main ideas of a subject (Cuando estoy estudiando pierdo bastante el tiempo con información irrelevante, antes de centrarme en las ideas principales de un tema)	Work Disconnection
*Item18*	*When I interrupt studying to rest of for any other reason, it takes me a while to get back on task* *(Cuando interrumpo mi estudio para descansar o por cualquier otro motivo, tardo en volver a ponerme a la tarea)*	*----*

r: reverse item; In parentheses item used in the study, Items removed from final solution in grey and italics, Items 1 to 7: Low work discipline, subscale of Study Problems Questionnaire (SPQ); item 8: adapted from Decisional Procrastination questionnaire (DP); items 11 and 13 adapted from subscale Procrastination from Academic Procrastination State Inventory (APSI; Schouwenburg); and items 14 and 16: adapted from General Procrastination Scale (GPS: Lay). Items 9, 10, 12, 15, 17 and 18: own creation (González-Brignardello and Sánchez-Elvira Paniagua).
